# Temporal Trends in Food Insecurity (Hunger) among School-Going Adolescents from 31 Countries from Africa, Asia, and the Americas

**DOI:** 10.3390/nu15143226

**Published:** 2023-07-20

**Authors:** Lee Smith, Guillermo F. López Sánchez, Mark A. Tully, Louis Jacob, Karel Kostev, Hans Oh, Laurie Butler, Yvonne Barnett, Jae Il Shin, Ai Koyanagi

**Affiliations:** 1 Centre for Health Performance and Wellbeing, Anglia Ruskin University, Cambridge CB1 1PT, UK; lee.smith@aru.ac.uk (L.S.); laurie.butler@aru.ac.uk (L.B.); yvonne.barnett@aru.ac.uk (Y.B.); 2 Division of Preventive Medicine and Public Health, Department of Public Health Sciences, School of Medicine, University of Murcia, 30120 Murcia, Spain; 3 School of Medicine, Ulster University, Londonderry BT48 7JL, UK; m.tully@ulster.ac.uk; 4 Research and Development Unit, Parc Sanitari Sant Joan de Déu, CIBERSAM, ISCIII, Dr. Antoni Pujadas, Sant Boi de Llobregat, 08830 Barcelona, Spain; louis.jacob.contacts@gmail.com (L.J.); koyanagi1117@gmail.com (A.K.); 5 Department of Physical and Rehabilitation Medicine, Lariboisière-Fernand Widal Hospital, AP-HP, University Paris Cité, 75010 Paris, France; 6 University Clinic of Marburg, 35037 Marburg, Germany; karel.kostev@iqvia.com; 7 Suzanne Dworak Peck School of Social Work, University of Southern California, Los Angeles, CA 90007, USA; hansoh@usc.edu; 8 Department of Pediatrics, Yonsei University College of Medicine, Seoul 03722, Republic of Korea; 9 ICREA, Pg. Lluis Companys 23, 08010 Barcelona, Spain

**Keywords:** food insecurity, hunger, temporal trends, adolescents, multi-country, epidemiology

## Abstract

(1) Background: Temporal trends of food insecurity among adolescents are largely unknown. Therefore, we aimed to examine this trend among school-going adolescents aged 12–15 years from 31 countries in Africa, Asia, and the Americas. (2) Methods: Data from the Global School-based Student Health Survey 2003–2017 were analyzed in 193,388 students [mean (SD) age: 13.7 (1.0) years; 49.0% boys]. The prevalence and 95%CI of moderate (rarely/sometimes hungry), severe (most of the time/always hungry), and any (moderate or severe) food insecurity (past 30-day) was calculated for each survey. Crude linear trends in food insecurity were assessed by linear regression models. (3) Results: The mean prevalence of any food insecurity was 52.2% (moderate 46.5%; severe 5.7%). Significant increasing and decreasing trends of any food insecurity were found in seven countries each. A sizeable decrease and increase were observed in Benin (71.2% in 2009 to 49.2% in 2016) and Mauritius (25.0% in 2011 to 43.6% in 2017), respectively. Severe food insecurity increased in countries such as Vanuatu (4.9% in 2011 to 8.4% in 2016) and Mauritius (3.5% in 2011 to 8.2% in 2017). The rate of decrease was modest in most countries with a significant decreasing trend, while many countries with stable trends showed consistently high prevalence of food insecurity. (4) Conclusion: Global action is urgently required to address food insecurity among adolescents, as our data show that achieving the United Nations Sustainable Development Goal 2 to end hunger and all forms of malnutrition by 2030 would be difficult without strong global commitment.

## 1. Introduction

Food insecurity may be defined as “limited or uncertain availability of nutritionally adequate and safe foods or limited or uncertain ability to acquire food in socially acceptable ways” [1]. Hunger is a related concept and is an uncomfortable or painful physical sensation that can be due to severe food insecurity. Undernourishment (an indicator of hunger) occurs in 10.8% of the global population, with rates varying from 5.5% in South America to 30.8% in Eastern Africa. Furthermore, approximately 828 million people were affected by hunger in 2021—46 million people more from 2020, and 150 million more from 2019 [2]. Importantly, the phenomenon of food insecurity is not unique to low- and middle-income countries (LMICs); it is also prevalent in high-income countries (HICs) such as the USA [3]. This highlights the challenge and difficulty in achieving the Sustainable Development Goal (SDG) 2 set by the United Nations to end hunger and all forms of malnutrition by 2030 [4].

Food insecurity, particularly among adolescents, is a public health concern, as it is associated with a plethora of detrimental outcomes [1,5]. For example, adolescents who experience food insecurity are at higher risk of mental health and psychosocial problems (e.g., social behavioral problems and worse academic performance) [1,6,7], overweight and obesity [8,9], and risky health behaviors (e.g., risky sexual activity [as a means to acquire food] and substance use) [10,11]. Moreover, hunger in youth increases the risk of chronic disease in adulthood, including, for example, diabetes and osteoporosis [12,13].

Improving our understanding of food insecurity is more important now than ever, as the COVID-19 pandemic has exacerbated food insecurity globally, interrupting many programs that promote well-being among adolescents (e.g., schools and school meals programs). The COVID-19 pandemic and other global challenges such as social unrest and environmental disasters compound existing vulnerabilities, such that the greatest challenges accrue to adolescents who have access to the fewest resources to promote their healthy development within their households, communities, and countries [14]. It is thus essential to understand the prevalence and temporal trends of food insecurity among adolescents to combat food insecurity in this population and to understand where we stand in the pursuit to achieve SDG 2. However, there is only one study on the temporal trends of food insecurity among adolescents, which reported the US trends in measures of food insecurity. Specifically, this study found that between 2007 and 2008, both the fraction of children/adolescents in food-insecure households and the rate of food insecurity among children/adolescents rose by one-third across those two years, and the rate of very low food security among children/adolescents increased by two-thirds, from 0.9 percent in 2007 to 1.5 percent in 2008, and after 2008, levels of these parameters remained high and stable [15]. Importantly, in general, there is limited literature on adolescent food insecurity outside the United States, raising uncertainty about the extent to which current thinking about food insecurity is specific to the social construction of adolescence in the United States [14]. More data are needed to understand the temporal trends in food insecurity among the global adolescent population. This is especially so for LMICs, where the prevalence of food insecurity has been reported to be high [16]. Moreover, conducting multi-country studies using standard questionnaires across countries on this topic is important, as it can allow for comparison between countries and provide insights on the reasons why some countries fare better or worse than others.

Given this background, the aim of the present study was to examine the temporal trends of food insecurity in a sample of 193,388 students aged 12–15 years from 31 countries in Africa, Asia, and the Americas (predominantly LMICs), where temporal trends of food insecurity are largely unknown.

## 2. Methods

Data from the Global School-based Student Health Survey (GSHS) were analyzed. Details on this survey can be found at https://www.who.int/teams/noncommunicable-diseases/surveillance/data and http://www.cdc.gov/gshs (accessed on 20 June 2023). Briefly, the GSHS was jointly developed by the WHO and the US Centers for Disease Control and Prevention (CDC), and other UN allies. The main aim of this survey was to examine and quantify risk and protective factors of major non-communicable diseases. The survey used a standardized two-stage probability sampling design for the selection process within each participating country. For the first stage, schools were selected with probability proportional to size sampling. The second stage involved the random selection of classrooms, which included students aged 13–15 years within each selected school. All students in the selected classrooms were eligible to participate in the survey regardless of age. Thus, the sample could have included adolescents who were not 13–15 years old. Data collection was performed during one regular class period. The questionnaire was translated into the local language in each country and consisted of multiple-choice response options. Students recorded their response on computer scannable sheets. All GSHS surveys were approved, in each country, by both a national government administration (most often the Ministry of Health or Education) and an institutional review board or ethics committee. Student privacy was protected through anonymous and voluntary participation, and informed consent was obtained as appropriate from the students, parents, and/or school officials. Data were weighted for non-response and probability selection.

From all publicly available data, we selected all nationally representative datasets that included the variables pertaining to our analysis and for which data on at least two waves were available from the same country. A total of 31 countries were included in the current study. The characteristics of each country, including the survey year, country income level, response rate, and sample size, are provided in Table A1 of Appendix A. The surveys included in the current study were conducted between 2003 and 2017, and were mainly from LMICs.

### 2.1. Food Insecurity (Hunger)

Food insecurity (hunger) was assessed by the question, “During the past 30 days, how often did you go hungry because there was not enough food in your home?” Answer options were categorized as no food insecurity (never), moderate food insecurity (rarely/sometimes), and severe food insecurity (most of the time/always) [17]. We named these categories as such since moderate food insecurity is often considered to be an indication that quality/quantity of food consumed has been compromised, while severe food insecurity refers to reduced food intake and disrupted eating patterns [18]. Any food insecurity referred to both moderate and severe food insecurity.

### 2.2. Statistical Analysis

Statistical analyses were performed with Stata 14.2 (Stata Corp LP, College Station, TX, USA). The analysis only included those aged 12–15 years, as most students were within this age group, while information on the exact age outside of this age range was not available. The prevalence and 95% CI of any food insecurity, moderate food insecurity, and severe food insecurity were calculated for each survey. Crude linear trends in any food insecurity, moderate food insecurity, and severe food insecurity were assessed by linear regression models across surveys within the same country to estimate regression coefficients (beta) and 95% CI for every one-year change. *p* for trends were estimated using the survey year as a continuous variable. Sampling weights and the clustered sampling design of the surveys were taken into account in all analyses.

## 3. Results

Data on 193,388 students aged 12–15 years [mean (SD) age 13.7 (1.0) years; 49.0% boys] were analyzed. Across all surveys, the mean prevalence of any food insecurity was 52.2%, while that of moderate and severe food insecurity were 46.5% and 5.7%, respectively. At the individual survey level, the prevalence of any food insecurity ranged from 18.7% (Uruguay in 2006) to 81.2% (Samoa in 2011). High prevalence of severe food insecurity was observed in Samoa in 2011 (36.0%), Benin in 2009 (19.0%), Seychelles in 2007 (16.6%), and Yemen in 2008 (15.4%).

**Figure 1:**
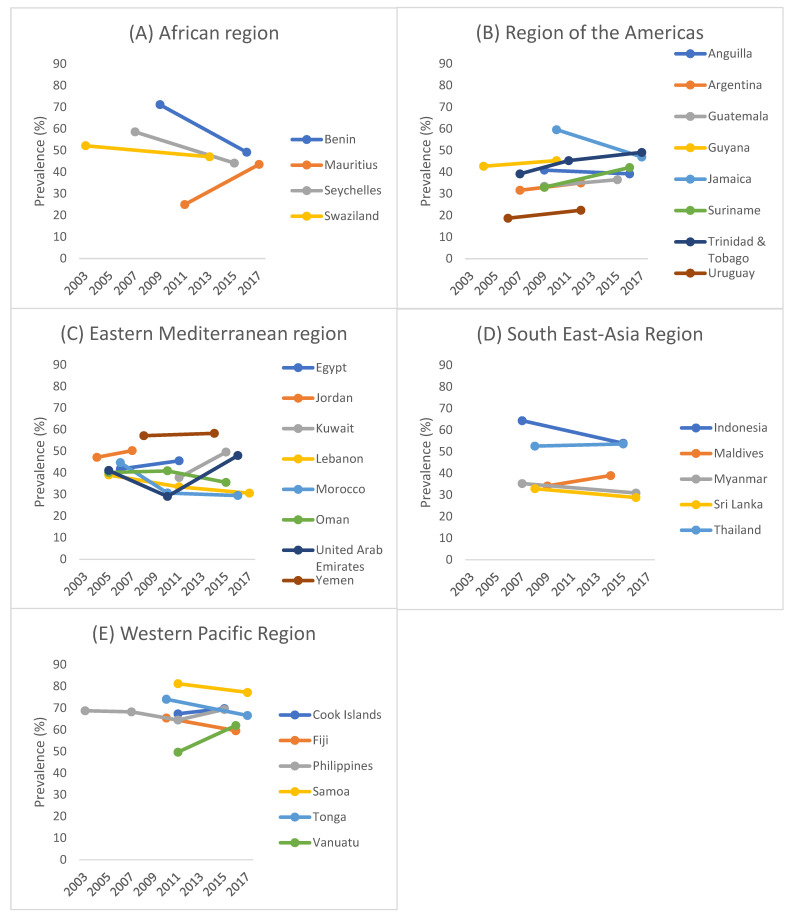
Trends in prevalence of any food insecurity by country and region.

The trends in prevalence of any food insecurity are shown in Table 1 and Figure 1. Of the 31 countries included in the study, significant increasing and decreasing trends of any food insecurity were found in seven countries each. Specifically, significant increasing trends were found in: Mauritius between 2011 (25.0%) and 2017 (43.6%) (beta = 3.09; 95%CI = 2.16, 4.03), Suriname between 2009 (33.0%) and 2016 (42.1%) (beta = 1.30; 95%CI = 0.37, 2.23), Trinidad & Tobago between 2007 (39.2%) and 2017 (49.1%) (beta = 0.97; 95%CI = 0.39, 1.55), Uruguay between 2006 (18.7%) and 2021 (22.4%) (beta = 0.61; 95%CI = 0.15, 1.08), Kuwait between 2011 (37.8%) and 2015 (49.6%) (beta = 2.95; 95%CI = 1.76, 4.15), United Arab Emirates between 2005 (41.1%) and 2016 (48.0%) (beta = 0.76; 95%CI = 0.34, 1.18), and Vanuatu between 2011 (49.7%) and 2016 (62.0%) (beta = 2.47; 95%CI = 0.73, 4.21). The beta can be interpreted as the average point change in prevalence (%) per year. On the other hand, significant decreasing trends were found in: Benin between 2009 (71.2%) and 2016 (49.2%) (beta = −3.14; 95%CI = −4.61, −1.66), Seychelles between 2007 (58.6%) and 2015 (44.2%) (beta = −1.80; 95%CI = −2.21, −1.39), Jamaica between 2010 (59.6%) and 2017 (47.0%) (beta = −1.79; 95%CI = −2.79, −0.80), Lebanon between 2005 (39.0%) and 2017 (30.6%) (beta = −0.70; 95%CI = −1.00, −0.39), Morocco between 2006 (44.7%) and 2016 (29.5%) (beta = −1.35; 95%CI = −1.74, −0.95), Indonesia between 2007 (64.4%) and 2015 (53.9%) (beta = −1.31, 95%CI = −1.95, −0.67), and Tonga between 2010 (74.1%) and 2017 (66.6%) (beta = −1.06; 95%CI = −1.61, −0.51). No significant increasing or decreasing trends were found in the remaining 17 countries.

**Figure 2:**
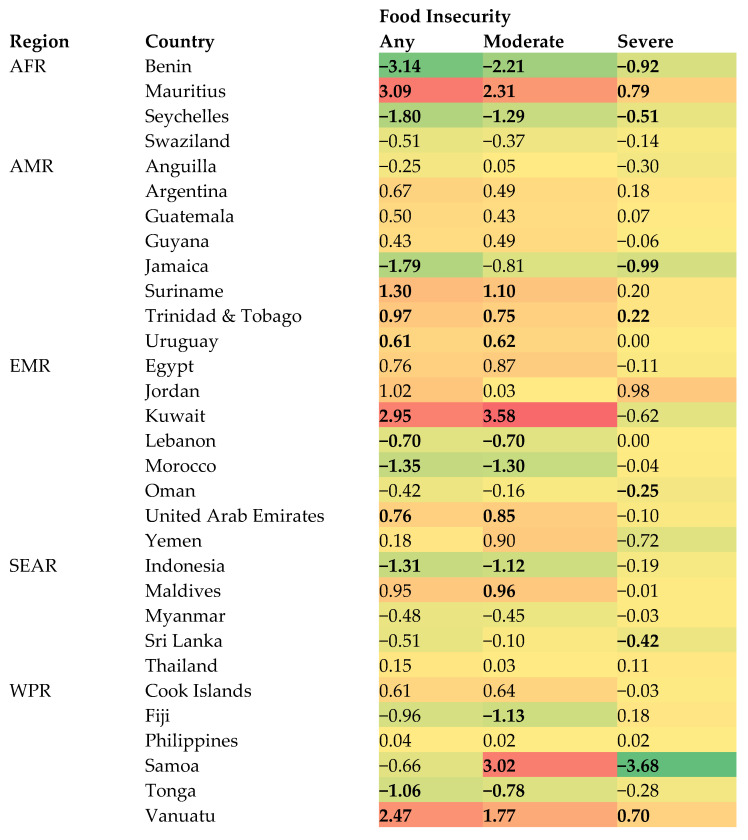
Heat map of the average percentage point change in prevalence per year. Abbreviation: AFR African region; AMR region of the Americas; EMR Eastern Mediterranean region; SEAR South-East Asia region; WPR Western Pacific region. The estimates correspond to the beta coefficient based on linear regression including survey year as a continuous variable. Bold fonts signify statistical significance (*p* < 0.05). Warmer colors indicate higher values and colder colors indicate lower values. Red is the warmest color and purple is the coldest color.

The trends in the prevalence of moderate and severe food insecurity are shown in Table 2, and the average percentage point change in prevalence of any, moderate, and severe food insecurity is visually displayed in Figure 2. While the directions of the trend were the same for any, moderate, and severe food insecurity in most countries, in countries such as Lebanon, Morocco, Indonesia, and Tonga, a significant decreasing trend was only observed for moderate food insecurity and not for severe food insecurity. Furthermore, in Samoa, even though there were no significant trends based on any food insecurity, there was a significant increase in moderate food insecurity but a significant decrease in severe food insecurity. Finally, the significant increasing trend of any food insecurity in Kuwait was largely explained by increase in moderate food insecurity but not severe food insecurity.

## 4. Discussion

### 4.1. Main Findings

In the present study, including large representative samples of school-going adolescents aged 12–15 years from 31 countries in Africa, Asia, and the Americas, significant increasing and decreasing trends of any food insecurity were observed in seven countries each. Mauritius observed the greatest increasing trend [2011 (25.0%) and 2017 (43.6%)], whereas Benin experienced the greatest decreasing trend [2009 (71.2%) and 2016 (49.2%)]. The rate of decrease was modest in the majority of countries with a significant declining trend. No significant changes in trends were observed in 17 countries, but it is important to note that levels of food insecurity in the majority of these countries were high across multiple years. When considering moderate and severe food insecurity, the direction of the trend was the same for the majority of countries, with some nuances observed in seven countries. To the best of the authors’ knowledge, this is the first global study to examine trends in food insecurity among adolescents across multiple continents, while it is the first to include data from LMICs.

### 4.2. Interpretation of the Findings

The fact that decreasing trends in food insecurity were observed in seven countries is encouraging. Such decreasing trends may have been achieved from country-wide initiatives and/or policies to combat food insecurity. For example, in Benin, in 2015, the World Food Programme was implemented to support and enhance existing initiatives. Benin considers nutrition at the center of development and utilizes nutrition-specific or related interventions through a multi-sectorial approach via its Strategic Plan for Food and Nutrition Development, with an emphasis on the implementation of community-level nutrition activities. In addition, the Plan for Development of the Education Sector highlights the importance of school meals to ameliorate retention rates. The government’s policy on school meals involves a multi-sector approach connecting local food production, nutrition, and education, and the national school meals policy’s long-term goal is to guarantee school meals for all Beninese schoolchildren [19]. However, an equal number of countries (n = 7) observed a significant increasing trend in food insecurity. A rise in an already high level of food insecurity is of upmost concern and may be explained by socio-economic hardship and lack of governmental support to address such issues. For example, in Mauritius, prices for staple food items have been increasing for the past 10 years, and less than 25% of its food products are produced locally. Moreover, in some areas of Mauritius, there is severely limited access to food products. For instance, the villages of Tamarin, Rivière Noire, and Petite Rivière Noire have access to only one vegetable seller. For fruits and vegetables, inhabitants of the region rely mainly on two supermarkets, which commercially target only middle- to upper-income customers. In this context, low-income households face challenges to have a nutritionally diversified and balanced diet, and importantly, this situation is common in other parts of the country [20].

Finally, our study also highlights the importance of assessing food insecurity in terms of severity. For instance, in countries such as Lebanon, Morocco, Indonesia, and Tonga, although there was a significant decreasing trend for any food insecurity, there was no significant decreasing trend for severe food insecurity. Furthermore, in Samoa, even though there was no significant trend for any food insecurity, there was a significant increasing trend for severe food insecurity. While the reasons for these findings are unclear, this may be related to the degree of inequality or the difficulty to reach the poorest or the most vulnerable segment within a country. For example, since 2005, Lebanon has been characterized by extreme inequality in both income and wealth. The richest one percent of the population receives, on average, 25 percent of the national income, while the poorest half receives less than 10 percent [21]. Given that the consequences of severe food insecurity are likely to be particularly severe, these data highlight the importance of considering severity of food insecurity when analyzing trends, as focusing solely on any food insecurity may mask important trends.

### 4.3. Policy Implications

Our findings underscore the importance of adopting a multi-dimensional poverty lens, as people can experience major deprivations irrespective of household income. Food insecurity remains a key indicator of well-being and may be developmentally impactful on children, adolescents, and young people, who are not financially independent. According to the World Food Programme, 135 million people suffer from acute hunger, owing to man-made conflicts, climate change, and economic downturns, with a greater impact now expected due to the COVID-19 pandemic [22]. Our data also suggest that there is a long way to achieve the United Nations SDG 2, which has as an aim “Zero Hunger by 2030”. While there are general strategies to reduce food insecurity that apply to the entire population (e.g., increasing agricultural productivity, investment in rural infrastructure [22]), there are some strategies which are aimed at reducing food insecurity specifically among school-going adolescents. For example, a common response to food insecurity is means-tested or universal social programming implemented in school settings, and may include initiatives such as free school meals, breakfast clubs, and school-operated food banks. However, it is important to highlight that such initiatives require either strong political or community buy-in [23].

### 4.4. Strengths and Limitations

The large representative sample of school-going adolescents from 31 countries, and the use of standard methodology across surveys are clear strengths of the present study. However, findings must be interpreted in light of the study’s limitations. First, food insecurity was self-reported, potentially introducing some level of bias (e.g., recall bias, social desirability bias) into the findings. Second, our study results are only generalizable to school-going adolescents, as only students were included in the study. However, it is worth noting that school attendance rates are known to be generally high in the countries included in our study. Finally, surveys were undertaken in different years depending on the country, and more data points were available in some countries than others. Thus, the beta coefficients estimated in our study are not totally comparable across countries, and they should always be interpreted in conjunction with the year in which the surveys were conducted.

## 5. Conclusions

In the present study including large representative samples of school-going adolescents aged 12–15 years from 31 countries in Africa, Asia, and the Americas, we observed a generally high level of food insecurity, with significant increasing and decreasing trends being observed in seven countries each. Global action is required to address food insecurity among adolescents, as our data reinforce the notion that the world is unlikely to be on track to achieve the SDG 2, which has as a goal to end hunger by 2030. It thus may be prudent to implement means-tested or universal social programming in school settings, and this may include initiatives such as free school meals, breakfast clubs, and school-operated food banks.

## Figures and Tables

**Table 1:** Trends in prevalence (%) of any food insecurity in 31 countries.

Country	Year	%	[95%CI]	Beta ^a^	[95%CI]	*p* for Trend ^a^	Country	Year	%	[95%CI]	Beta ^a^	[95%CI]	*p* for Trend ^a^
**AFR**							EMR (continued)						
Benin	2009	71.2	[66.4, 75.6]	−3.14	[−4.61, −1.66]	<0.001	Morocco	2006	44.7	[41.7, 47.8]	−1.35	[−1.74, −0.95]	<0.001
	2016	49.2	[40.5, 58.1]					2010	30.7	[26.8, 34.9]			
Mauritius	2011	25.0	[21.6, 28.8]	3.09	[2.16, 4.03]	<0.001		2016	29.5	[27.3, 31.8]			
	2017	43.6	[39.7, 47.6]				Oman	2005	40.2	[37.3, 43.1]	−0.42	[−0.86, 0.03]	0.066
Seychelles	2007	58.6	[57.9, 59.3]	−1.80	[−2.21, −1.39]	<0.001		2010	40.9	[36.9, 45.1]			
	2015	44.2	[41.0, 47.4]					2015	35.6	[32.3, 38.9]			
Swaziland	2003	52.2	[49.8, 54.7]	−0.51	[−1.04, 0.01]	0.055	United Arab Emirates	2005	41.1	[39.7, 42.5]	0.76	[0.34, 1.18]	<0.001
	2013	47.1	[42.6, 51.7]					2010	29.1	[26.9, 31.4]			
**AMR**								2016	48.0	[43.9, 52.2]			
Anguilla	2009	40.9	[40.9, 40.9]	−0.25	[−0.89, 0.39]	0.432	Yemen	2008	57.2	[48.5, 65.5]	0.18	[−1.66, 2.02]	0.845
	2016	39.2	[34.9, 43.6]					2014	58.3	[52.0, 64.3]			
Argentina	2007	31.6	[28.2, 35.3]	0.67	[−0.08, 1.42]	0.081	**SEAR**						
	2012	35.0	[33.8, 36.1]				Indonesia	2007	64.4	[59.9, 68.6]	−1.31	[−1.95, −0.67]	<0.001
Guatemala	2009	33.5	[28.6, 38.7]	0.50	[−0.88, 1.89]	0.475		2015	53.9	[51.3, 56.4]			
	2015	36.5	[30.3, 43.2]				Maldives	2009	34.2	[30.3, 38.3]	0.95	[−0.19, 2.09]	0.101
Guyana	2004	42.7	[37.4, 48.2]	0.43	[−0.82, 1.69]	0.488		2014	39.0	[35.0, 43.0]			
	2010	45.3	[40.6, 50.1]				Myanmar	2007	35.3	[30.7, 40.1]	−0.48	[−1.10, 0.13]	0.122
Jamaica	2010	59.6	[55.3, 63.7]	−1.79	[−2.79, −0.80]	0.001		2016	30.9	[28.3, 33.6]			
	2017	47.0	[41.8, 52.3]				Sri Lanka	2008	32.9	[30.1, 35.8]	−0.51	[−1.11, 0.08]	0.088
Suriname	2009	33.0	[30.1, 36.0]	1.30	[0.37, 2.23]	0.008		2016	28.8	[25.3, 32.5]			
	2016	42.1	[36.7, 47.6]				Thailand	2008	52.6	[49.4, 55.7]	0.15	[−0.49, 0.78]	0.641
Trinidad & Tobago	2007	39.2	[34.7, 43.9]	0.97	[0.39, 1.55]	0.001		2015	53.6	[50.7, 56.6]			
	2011	45.3	[41.3, 49.3]				**WPR**						
	2017	49.1	[45.5, 52.6]				Cook Islands	2011	67.4	[67.4, 67.4]	0.61	[−0.45, 1.67]	0.251
Uruguay	2006	18.7	[16.9, 20.7]	0.61	[0.15, 1.08]	0.011		2015	69.8	[65.6, 73.8]			
	2012	22.4	[20.5, 24.4]				Fiji	2010	65.4	[59.3, 71.0]	−0.96	[−2.21, 0.29]	0.129
**EMR**								2016	59.6	[55.5, 63.7]			
Egypt	2006	41.8	[35.8, 48.1]	0.76	[−1.46, 2.97]	0.496	Philippines	2003	68.8	[65.2, 72.1]	0.04	[−0.27, 0.35]	0.799
	2011	45.6	[37.0, 54.6]					2007	68.3	[65.9, 70.7]			
Jordan	2004	47.2	[43.7, 50.8]	1.02	[−1.03, 3.06]	0.317		2011	64.5	[60.7, 68.1]			
	2007	50.3	[45.6, 54.9]					2015	69.4	[67.1, 71.7]			
Kuwait	2011	37.8	[34.9, 40.8]	2.95	[1.76, 4.15]	<0.001	Samoa	2011	81.2	[78.5, 83.6]	−0.66	[−1.49, 0.18]	0.119
	2015	49.6	[46.1, 53.2]					2017	77.2	[72.8, 81.1]			
Lebanon	2005	39.0	[36.9, 41.1]	−0.70	[−1.00, −0.39]	<0.001	Tonga	2010	74.1	[71.6, 76.4]	−1.06	[−1.61, −0.51]	<0.001
	2011	33.5	[30.5, 36.6]					2017	66.6	[63.6, 69.5]			
	2017	30.6	[27.7, 33.6]				Vanuatu	2011	49.7	[42.3, 57.1]	2.47	[0.73, 4.21]	0.006
								2016	62.0	[57.8, 66.0]			

Abbreviation: CI confidence interval; AFR African region; AMR region of the Americas; EMR Eastern Mediterranean region; SEAR South-East Asia region; WPR Western Pacific region. ^a^ The beta and *p* for trend are based on linear regression including survey year as a continuous variable. The beta can be interpreted as the average percentage point change in prevalence per year.

**Table 2:** Trends in prevalence of moderate food insecurity and severe food insecurity in 31 countries.

		Moderate Food Insecurity					Severe Food Insecurity				
Country	Year	%	[95%CI]	Beta	[95%CI]	*p* for Trend	%	[95%CI]	Beta	[95%CI]	*p* for Trend
**AFR**											
Benin	2009	52.2	[48.3, 56.2]	−2.21	[−3.35, −1.08]	<0.001	19.0	[14.7, 24.2]	−0.92	[−1.83, −0.02]	0.044
	2016	36.7	[30.5, 43.5]				12.5	[9.2, 16.8]			
Mauritius	2011	21.5	[18.5, 25.0]	2.31	[1.50, 3.11]	<0.001	3.5	[2.7, 4.5]	0.79	[0.25, 1.33]	0.006
	2017	35.4	[32.2, 38.7]				8.2	[5.7, 11.7]			
Seychelles	2007	41.9	[41.2, 42.6]	−1.29	[−1.64, −0.95]	<0.001	16.6	[16.1, 17.2]	−0.51	[−0.81, −0.20]	0.001
	2015	31.6	[29.0, 34.3]				12.6	[10.4, 15.1]			
Swaziland	2003	42.8	[40.8, 44.8]	−0.37	[−0.82, 0.07]	0.096	9.4	[8.3, 10.6]	−0.14	[−0.39, 0.11]	0.282
	2013	39.1	[35.3, 43.0]				8.0	[6.1, 10.6]			
**AMR**											
Anguilla	2009	33.9	[33.9, 33.9]	0.05	[−0.63, 0.72]	0.891	7.0	[7.0, 7.0]	−0.30	[−0.61, 0.01]	0.058
	2016	34.2	[29.8, 38.9]				4.9	[3.2, 7.5]			
Argentina	2007	29.0	[25.3, 33.0]	0.49	[−0.31, 1.29]	0.227	2.6	[1.6, 4.2]	0.18	[−0.10, 0.46]	0.216
	2012	31.5	[30.5, 32.5]				3.5	[2.9, 4.2]			
Guatemala	2009	31.2	[26.6, 36.3]	0.43	[−0.87, 1.74]	0.511	2.3	[1.7, 3.0]	0.07	[−0.13, 0.27]	0.507
	2015	33.8	[28.1, 40.0]				2.7	[1.8, 3.9]			
Guyana	2004	34.3	[31.8, 37.0]	0.49	[−0.32, 1.30]	0.227	8.4	[5.2, 13.1]	−0.06	[−0.87, 0.75]	0.882
	2010	37.3	[33.4, 41.3]				8.0	[5.7, 11.1]			
Jamaica	2010	46.4	[40.3, 52.6]	−0.81	[−1.95, 0.34]	0.161	13.1	[9.7, 17.5]	−0.99	[−1.62, −0.35]	0.003
	2017	40.8	[36.2, 45.5]				6.2	[4.6, 8.4]			
Suriname	2009	24.8	[22.5, 27.3]	1.10	[0.30, 1.89]	0.009	8.1	[6.4, 10.2]	0.20	[−0.14, 0.54]	0.234
	2016	32.5	[28.0, 37.4]				9.5	[8.4, 10.9]			
Trinidad & Tobago	2007	33.2	[29.1, 37.4]	0.75	[0.20, 1.29]	0.008	6.0	[4.5, 8.1]	0.22	[0.01, 0.43]	0.036
	2011	39.0	[35.3, 42.8]				6.3	[5.2, 7.7]			
	2017	40.9	[37.3, 44.5]				8.2	[7.1, 9.5]			
Uruguay	2006	17.2	[15.5, 19.1]	0.62	[0.17, 1.06]	0.007	1.5	[1.1, 2.0]	0.00	[−0.11, 0.10]	0.949
	2012	20.9	[19.1, 22.9]				1.5	[1.1, 1.9]			
**EMR**											
Egypt	2006	36.8	[31.0, 43.0]	0.87	[−1.35, 3.09]	0.435	5.1	[3.8, 6.8]	−0.11	[−0.58, 0.35]	0.622
	2011	41.1	[32.5, 50.4]				4.5	[3.1, 6.5]			
Jordan	2004	37.0	[34.9, 39.2]	0.03	[−1.49, 1.56]	0.964	10.2	[8.5, 12.3]	0.98	[−0.02, 1.99]	0.055
	2007	37.1	[33.4, 41.0]				13.2	[11.1, 15.5]			
Kuwait	2011	28.9	[26.3, 31.6]	3.58	[2.60, 4.56]	<0.001	8.9	[7.3, 10.9]	−0.62	[−1.35, 0.10]	0.089
	2015	43.2	[40.6, 45.9]				6.4	[4.6, 8.9]			
Lebanon	2005	36.0	[34.0, 38.0]	−0.70	[−0.98, −0.41]	<0.001	3.0	[2.6, 3.6]	0.00	[−0.07, 0.07]	0.951
	2011	29.9	[27.3, 32.6]				3.6	[2.5, 5.1]			
	2017	27.5	[24.9, 30.2]				3.1	[2.5, 3.8]			
Morocco	2006	35.7	[33.3, 38.1]	−1.30	[−1.71, −0.90]	<0.001	9.0	[7.2, 11.4]	−0.04	[−0.30, 0.22]	0.741
	2010	21.0	[17.5, 24.9]				9.7	[8.5, 11.1]			
	2016	20.7	[18.0, 23.7]				8.8	[7.3, 10.5]			
Oman	2005	32.8	[30.0, 35.6]	−0.16	[−0.60, 0.27]	0.453	7.4	[6.2, 8.7]	−0.25	[−0.43, −0.08]	0.006
	2010	32.0	[28.7, 35.4]				8.9	[7.0, 11.3]			
	2015	31.1	[28.0, 34.5]				4.4	[3.4, 5.7]			
United Arab Emirates	2005	32.1	[30.9, 33.4]	0.85	[0.47, 1.24]	<0.001	8.9	[8.2, 9.7]	−0.10	[−0.23, 0.04]	0.163
	2010	24.3	[22.8, 25.8]				4.8	[3.6, 6.4]			
	2016	40.5	[36.7, 44.3]				7.5	[6.4, 8.9]			
Yemen	2008	41.8	[36.2, 47.6]	0.90	[−0.63, 2.42]	0.240	15.4	[10.3, 22.4]	−0.72	[−1.82, 0.38]	0.192
	2014	47.2	[40.6, 53.9]				11.1	[9.2, 13.3]			
**SEAR**											
Indonesia	2007	58.7	[55.0, 62.2]	−1.12	[−1.69, −0.56]	<0.001	5.7	[4.0, 8.0]	−0.19	[−0.45, 0.07]	0.157
	2015	49.7	[47.1, 52.2]				4.2	[3.6, 4.9]			
Maldives	2009	28.1	[25.4, 31.1]	0.96	[0.03, 1.90]	0.044	6.1	[4.4, 8.3]	−0.01	[−0.49, 0.47]	0.966
	2014	32.9	[29.4, 36.7]				6.0	[4.8, 7.6]			
Myanmar	2007	32.4	[28.2, 36.9]	−0.45	[−1.00, 0.10]	0.107	2.9	[1.8, 4.6]	−0.03	[−0.25, 0.18]	0.745
	2016	28.4	[26.3, 30.5]				2.5	[1.5, 4.2]			
Sri Lanka	2008	26.2	[23.2, 29.4]	−0.10	[−0.71, 0.52]	0.749	6.7	[5.7, 7.9]	−0.42	[−0.59, −0.25]	<0.001
	2016	25.4	[22.0, 29.2]				3.4	[2.7, 4.2]			
Thailand	2008	49.1	[46.3, 52.0]	0.03	[−0.52, 0.59]	0.900	3.5	[2.7, 4.5]	0.11	[−0.08, 0.30]	0.237
	2015	49.4	[46.9, 51.9]				4.3	[3.4, 5.3]			
**WPR**											
Cook Islands	2011	59.0	[59.0, 59.0]	0.64	[−0.79, 2.07]	0.372	8.4	[8.4, 8.4]	−0.03	[−0.87, 0.81]	0.943
	2015	61.6	[55.9, 66.9]				8.3	[5.5, 12.2]			
Fiji	2010	54.9	[50.2, 59.5]	−1.13	[−2.19, −0.08]	0.036	10.5	[8.6, 12.7]	0.18	[−0.32, 0.67]	0.467
	2016	48.1	[44.2, 52.0]				11.6	[9.7, 13.7]			
Philippines	2003	61.5	[58.5, 64.5]	0.02	[−0.24, 0.28]	0.880	7.2	[6.0, 8.6]	0.02	[−0.12, 0.16]	0.775
	2007	61.8	[59.6, 64.0]				6.5	[5.7, 7.5]			
	2011	57.8	[54.1, 61.4]				6.6	[5.5, 8.1]			
	2015	62.2	[60.3, 64.0]				7.2	[6.1, 8.5]			
Samoa	2011	45.2	[41.3, 49.1]	3.02	[1.99, 4.05]	<0.001	36.0	[33.7, 38.3]	−3.68	[−4.46, −2.90]	<0.001
	2017	63.3	[58.6, 67.8]				13.9	[10.4, 18.3]			
Tonga	2010	60.3	[57.5, 63.1]	−0.78	[−1.34, −0.22]	0.007	13.7	[12.0, 15.7]	−0.28	[−0.66, 0.10]	0.149
	2017	54.9	[52.1, 57.6]				11.8	[10.0, 13.8]			
Vanuatu	2011	44.7	[38.2, 51.5]	1.77	[0.04, 3.49]	0.045	4.9	[3.3, 7.2]	0.70	[0.17, 1.23]	0.011
	2016	53.6	[48.4, 58.6]				8.4	[6.8, 10.4]			

Abbreviation: CI confidence interval; AFR African region; AMR region of the Americas; EMR Eastern Mediterranean region; SEAR South-East Asia region; WPR Western Pacific region. The beta and *p* for trend are based on linear regression including survey year as a continuous variable. The beta can be interpreted as the average percentage point change in prevalence per year.

## Data Availability

The data presented in this study are available on request from the corresponding author.

## Appendix A

**Table A1:** Survey characteristics.

Region	Country	Country Income	Year	Response Rate (%)	N ^a^
AFR	Benin	L	2009	90	1170
		L	2016	78	717
	Mauritius	UM	2011	82	2074
		UM	2017	84	1955
	Seychelles	UM	2007	82	1154
		H	2015	82	2061
	Swaziland	LM	2003	96	6866
		LM	2013	97	1318
AMR	Anguilla	NA	2009	84	701
		NA	2016	88	564
	Argentina	UM	2007	77	1537
		UM	2012	71	21,528
	Guatemala	LM	2009	81	4495
		LM	2015	82	3611
	Guyana	LM	2004	80	1070
		LM	2010	76	1973
	Jamaica	UM	2010	72	1204
		UM	2017	60	1061
	Suriname	UM	2009	89	1046
		UM	2016	83	1453
	Trinidad & Tobago	H	2007	78	2450
		H	2011	90	2363
		H	2017	89	2763
	Uruguay	UM	2006	71	2882
		H	2012	77	2869
EMR	Egypt	LM	2006	87	4981
		LM	2011	85	2364
	Jordan	LM	2004	95	1848
		LM	2007	99.8	1648
	Kuwait	H	2011	85	2298
		H	2015	78	2034
	Lebanon	UM	2005	88	4524
		UM	2011	87	1982
		UM	2017	82	3347
	Morocco	LM	2006	84	1986
		LM	2010	92	2405
		LM	2016	91	3975
	Oman	UM	2005	97	2426
		H	2010	89	1000
		H	2015	92	1669
	United Arab Emirates	H	2005	89	12,819
		H	2010	91	2302
		H	2016	80	3471
	Yemen	L	2008	82	905
		LM	2014	75	1553
SEAR	Indonesia	LM	2007	93	3022
		LM	2015	94	8806
	Maldives	LM	2009	80	1981
		UM	2014	60	1781
	Myanmar	L	2007	95	2227
		LM	2016	86	2237
	Sri Lanka	LM	2008	89	2504
		LM	2016	89	2254
	Thailand	LM	2008	93	2675
		UM	2015	89	4132
WPR	Cook Islands	NA	2011	84	849
		NA	2015	65	366
	Fiji	LM	2010	90	1495
		UM	2016	79	1537
	Philippines	LM	2003	84	4198
		LM	2007	81	3484
		LM	2011	82	3845
		LM	2015	79	6162
	Samoa	LM	2011	79	2200
		LM	2017	59	1058
	Tonga	LM	2010	80	1946
		UM	2017	90	2067
	Vanuatu	LM	2011	72	852
		LM	2016	57	1288

^a^ Based on sample aged 12–15 years. Abbreviation: AFR African region; AMR region of the Americas; EMR Eastern Mediterranean region; SEAR South-East Asia region; WPR Western Pacific region; H high income; L low income; LM lower middle income; UM upper middle income. Data on country income were not available from Cook Islands and Anguilla.
